# Effects of Endosymbiont Disruption on the Nutritional Dynamics of the Pea Aphid *Acyrthosiphon pisum*

**DOI:** 10.3390/insects9040161

**Published:** 2018-11-10

**Authors:** Ning Lv, Lei Wang, Wen Sang, Chang-Zhong Liu, Bao-Li Qiu

**Affiliations:** 1 Key Laboratory of Bio-Pesticide Innovation and Application, South China Agricultural University, Guangzhou 510640, China; lning2013@126.com (N.L.); wangleiscau@163.com (L.W.); sangwen@scau.edu.cn (W.S.); 2 College of Plant Protection, Gansu Agricultural University, Lanzhou 730070, China; liuchzh@gsau.edu.cn; 3 Engineering Technology Research Center of Agricultural Pest Biocontrol, Guangzhou 510640, China

**Keywords:** *Buchnera*, pea aphid, endosymbiont, antibiotics, nutrition

## Abstract

Pea aphid (*Acyrthosiphon pisum*) is a worldwide pest that feeds exclusively on the phloem sap of numerous host plants. It harbours a well-known primary endosymbiont *Buchnera* *aphidicola* that helps to overcome the nutritional deficiency of a plant-based diet. However, how the *Buchnera* contributes to the nutritional and energy metabolism of its aphid host is unclear to date. In the current study, the function of *Buchnera* in relation to nutritional synthesis of pea aphid was investigated by disrupting the primary endosymbiont with an antibiotic rifampicin. Our findings revealed that the disruption of *Buchnera* led to infertility and higher loss in body mass of aphid hosts. Body length and width were also decreased significantly compared to healthy aphids. The detection of nutrition indicated that the quantity of proteins, soluble sugars, and glycogen in aposymbiotic pea aphids increased slowly with the growth of the aphid host. In comparison, the quantities of all the nutritional factors were significantly lower than those of symbiotic pea aphids, while the quantity of total lipid and neutral fat in aposymbiotic pea aphids were distinctly higher than those of symbiotic ones. Thus, we concluded that the significant reduction of the total amount of proteins, soluble sugars, and glycogen and the significant increase of neutral fats in aposymbiotic pea aphids were due to the disruption of *Buchnera*, which confirmed that the function of *Buchnera* is irreplaceable in the pea aphid.

## 1. Introduction

Bacterial symbionts are found in a diverse array of insects and play important roles in plant–insect interactions [1,2]. These symbionts usually fall into two categories: firstly, primary symbionts, which provide their host with essential nutrition, including amino acids and vitamins, in order to complete their diet. These symbionts are necessary for host survival and development [3,4]. Secondly, secondary symbionts, in contrast, are involved in reproductive manipulations. They have the ability to cope with environmental factors such as heat stress, chemical insecticides, and also aid in defense against parasitoids or pathogenic fungi [5,6,7,8]. Evidence of obligate endosymbionts influencing the host fitness of herbivorous insects has been demonstrated in several insect–obligate endosymbiont systems, such as the pea aphid–*Buchnera* [9,10,11], whitefly–*Portiera* [12,13,14], psyllid–*Carsonella* [15,16], and tsetse fly–*Wigglesworthia* [17]. However, the impact of obligate endosymbionts on nutritional metabolism, including protein, soluble sugar, glycogen, and lipids, in an herbivorous insect has been little studied. 

The pea aphid, *Acyrthosiphon pisum* Harris, is a worldwide pest that feeds on leguminous crops and forage grasses (such as peas, dry beans, alfalfa, clover, and fresh beans). Pea aphids, on entering a suitable host field, can very quickly cause economic injury because of their parthenogenic reproductive system and short generation times. As a result, serious production losses can occur [18,19]. Like other aphids, *A. pisum* feeds exclusively on the phloem sap of host plants, which is a diet rich in sugar but limited in essential amino acids and vitamins [20,21]. To overcome this nutritional deficiency, *A. pisum* harbours a primary endosymbiont, *Buchnera aphidicola*, which can provide them with the much-lacking essential amino acids and vitamins [10]. Essential amino acids could affect the growth and metabolism of pea aphids, such as tyrosine, phenylalanine, and lysine [22,23,24]. Moreover, the metabolic adjustments of the phloem-feeding insects to symbiont loss were broadly equivalent [25]. Furthermore, *B. aphidicola* is transmitted from one generation to the next generation through transovarial transmission [26]. Previous studies have revealed that disruption of *Buchnera* infection in aphids led to lower survival, slower development, and failure to produce offspring [27,28,29].

In addition to the primary endosymbiont, pea aphids also harbour several species of secondary endosymbionts, such as *Serratia*, *Hamiltonella*, and *Regiella*, all of which are involved in the biology of their host. In comparing the secondary endosymbionts to the primary endosymbiont, *Buchnera* is considered indispensable because of its role in nutrition provision and physical metabolism. However, the role that *Buchnera* play in the nutritional and energy metabolism of its aphid host is unclear to date. In the current study, we investigated the dynamic changes of nutrition, including protein, soluble sugar, glycogen, and lipids, in *A. pisum* by disrupting the primary endosymbiont *Buchnera* with an antibiotic. The aim of this study was to provide new and comprehensive insights into the function of *Buchnera* as well as its potential role in control strategies of pea aphids in agricultural systems.

## 2. Materials and Methods

### 2.1. Plants and Insects

Pea aphids, *A. pisum,* were initially collected from alfalfa plants in Lanzhou city, Gansu, China. A single asexual line was established for further experiments. The progenies of this asexual line were then reared on broad bean *Vicia fabae*, Lincan-9 in a greenhouse at 24 ± 1 °C, 70–80% relative humidity, and a photoperiod of 16:8 (L:D) for at least 10 generations before being used in experiments.

### 2.2. Antibiotic Screening for Endosymbiont Disruption in the Pea Aphid

Broad bean seeds were sown in plastic pots (Φ 15 cm) containing sand (30%), clay (30%), and nutritional soil (40%). At 6–8 expanded leaf stage, plant roots were cleaned with ddH_2_O and then individually placed into a triangle bottle filled with 150 mL of one of the six following antibiotic solutions: chlortetracycline HCl, chloraomycetin, oxytettracycline HCl, penicillin-GK salt, streptomycin sulfate, and rifampicin (all the antibiotics were from Sangon biotech, Shanghai, China). All the antibiotic solutions were 0.2 mg/mL. Each antibiotic treatment was repeated six times. Thirty wingless adults of pea aphid were introduced onto the individual bean plants to produce nymphs for 12 h. After 2 days of feeding on the bean plants treated with the antibiotic solutions, the new nymphal offspring were collected and released onto soil-potted healthy bean plants for continuous growth at 24 ± 1 °C, 70–80% relative humidity, and a photoperiod of 16:8 (L:D) within an insect growth chamber (RZX, Ningbo Jiangnan Co. Ltd., Ningbo, China). Double-distilled water was used as a root solution in control experiments. The experiment was repeated six times. 

The infections of primary endosymbiont *Buchnera* in the newly produced nymphal offspring were detected by PCR on the 6th and 11th day of age. The efficiencies of the six antibiotics to disrupt *Buchnera* functioning were compared. Ten aphids from each treatment and control sets were collected and washed with ddH_2_O. Then, each aphid was put into a 0.5-mL microcentrifuge tube containing 50 μL STE (10 mM Tris-HCl, pH 8.0, 25 mM NaCl, 25 mM EDTA, 1% SDS, proteinase K 200 mg/mL) for homogenization. The homogenate was incubated at 56 °C for 2–3 h and then placed in 95 °C water for 10 min to inactivate the proteinase K (Sangon biotech, Shanghai, China). After incubation, the samples were centrifuged for a short time and then used for PCR detection. PCR was conducted with the primers (forward, 5′-ATAATCGGTGGTGTTGGAGA-3′; reverse, 5′-AAGCAGCATATTGTAAAGCAGA-3′). Three biological replicates were performed for each antibiotic screen experiment, which means that 30 individuals in total were examined for antibiotic experiments.

The PCR procedure for *Buchnera* detection was as follows: firstly, pre-denaturation at 94 °C for 2 min, then followed by 35 cycles of 94 °C for 30 s, 51 °C for 30 s, and 72 °C for 60 s, and finally, a 10-min extension period at 72 °C. All PCRs were performed in a 25-μL reaction volume that included 2.5 mM MgCl_2_, 200 mM for each dNTPs, 1 μM of each primer, and 1 unit DNA Taq polymerase (Sangon biotech, Shanghai, China). After amplification, 7 μL of the PCR product was visualized on a 1% agarose gel containing GoldView colourant and then photographed. Each PCR detection included a negative control (ddH_2_O) and mitochondrial COI gene (The primers: LCO1490 (5′-CAACATTTATTTTGATTTTTTGG-3′) and HCO2198 (5′-CCTAAAAAATGTTGAGGGAAAAA-3′) was used as internal control for DNA quality of the samples. The results of PCR detection showed that rifampicin and oxytetracycline HCl were the optimal antibiotics for *Buchnera* disruption in pea aphids. Thus, hereafter, we used the experimental nymphs from the rifampicin treatments for further ecological and nutritional investigations.

### 2.3. Effects of Buchnera Disruption on the Growth of Pea Aphid

Pea aphid nymphs were prepared as per the above experiments. The number of treated and controlled aphids produced daily were recorded from the adult to the aphids dying and after 11 days of growth (including 2 days of feeding on the bean plants in the antibiotic solution, and then a 9-day growth period on untreated plants). Body sizes of the aphid nymphs from each treatment were measured using AxioVision SE64 Rel. 4.8 camera software with Zeiss postures microscopy. In addition, the bodies of the aphids from each experiment were also weighed using a high-precision (1/100,000) electronic balance. Ten pea aphids from each treatment and control set were measured with the investigations repeated three times. 

### 2.4. Quantification of the Dynamic Changes of Pea Aphid Nutrition

The nymphs of pea aphids were prepared as in the above experiments. Five pea aphids were randomly selected from both colonies of *Buchnera* infected and inactivated at days 2, 4, 6, 8, 10, and 12, respectively, to make 12 subsetups (six of *Buchnera* infected and six of inactivated), and each subsetup repeated was 6 times along with the aphid samples being weighed. The aphids were washed with ddH_2_O and placed into a 1.5-mL centrifuge tube containing 180 µL lysis buffer liquid (100 mM KH_2_PO_4_, 1 mM dithiothreitol, and 1 mM ethylenediaminetetraacetic acid, pH 7.4). The aphids were homogenized and then centrifuged at 16,000 rpm for 15 min at 4 °C, the homogenate of which was then used for further nutritional detection. Three replicates from each of the antibiotic-treated and control experimental sets were repeated for the various nutritional detections. Three biological replicates were performed for each biology sample. 

#### 2.4.1. Protein Detection

The protein content of the aphids in the different treatments was detected according to the method of Lowry et al. [30]. About 20 µL of the homogenate suspension was transferred into a 96-well coated plate and mixed with 200 µL of Coomassie brilliant blue for 15–20 min at room temperature. The optical density (OD) values were measured at 595 nm, and the protein contents were calculated based on the standard curve of bovine serum albumin (Sangon biotech, Shanghai, China). The experiment was repeated six times.

#### 2.4.2. Carbohydrate Detection

The soluble sugar and the glucogen contents of aphids from different treatments were detected according to the methods of van Handel [31] and van Handel and Day [32]. Briefly, 160 µL of the homogenate suspension was transferred into a 2-mL centrifuge tube and mixed with 20 µL of 20% sodium sulfate and 1500 µL of a chloroform/methanol solution (1:2 *v*/*v*). This mixture was then centrifuged at 10,000 rpm for 15 min at 4 °C for the following detections (hereafter, this mixture solution was treated as a premeasured solution). 

To detect the soluble sugars of an aphid, a 150-µL suspension of the premeasured solution was transferred into a 1.5-mL centrifuge tube and mixed with 10 µL ddH_2_O and 240 µL anthrone (1.42 g/L). The mixture was first incubated for 15 min at room temperature and then incubated in boiling water for a further 15 min, followed by cooling at room temperature. The mixture was transferred into a 96-pore coated plate. The OD value was measured at 630 nm, and the soluble sugar content was calculated based on the standard curve of D-glucose (Sangon biotech, Shanghai, China). 

The remaining suspension of the premeasured solution was transferred to another centrifuge tube. Here, the precipitant was mixed with 400 µL of 80% methanol and homogenized in an ultrasonic cleaning apparatus for 5–10 min, after which the homogenate was centrifuged again at 10,000 rpm for 15 min at 4 °C. The new suspension was then mixed with anthrone solution, incubated for 15 min at room temperature, and then in boiling water for a further 15 min. Finally, the mixture was transferred into a 96-pore coated plate. The OD value was measured at 630 nm, and the glucogen content was calculated based on the standard curve of D-glucose. The experiment was repeated six times.

#### 2.4.3. Lipid Detection

The total lipid and neutral fat of the aphids in the different treatments was detected according to the methods of van Handel [33] and Williams et al. [34]. To detect the total lipid content, 100 µL of the premeasured solution was transferred to a 1-mL centrifuge tube, then the solution was dried at 90 °C until all the solvents were completely evaporated. Following this, 10 µL of 98% sulphuric acid and 190 µL of vanillin solution (1.2 g/L) were added to the tube. After 15 min of incubation at room temperature, the liquid mixture was transferred into a 96-pore coated plate. The OD value of total lipid was measured at 525 nm, with the total lipid content was calculated based on the standard curve of triolein (Sigma, St. Louis, MO, USA). The experiment was repeated six times.

The method used for the detection of neutral fat in the aphid nymphs was similar to the method used for total lipid detection. Approximately 150 µL of the premeasured solution was transferred into a 1.5-mL centrifuge tube and then dried at 90 °C until all the solvents were completely evaporated. After that, 1 mL of chloroform was added to dissolve the solution content and then centrifuged at 10,000 rpm for 15 min at 4 °C. Following this, 100 µL of the new suspension was treated in the same procedure as for total lipid detection. The OD value of neutral lipid was measured at 525 nm, and its content was calculated based on the standard curve of triethylhexanoin (Aladdin, Shanghai, China). The experiment was repeated six times.

### 2.5. Statistical Analysis

The mean body size and weight and the content of nutritional reserves (including lipids, proteins, carbohydrates, and glycogen) of pea aphids in both *Buchnera* infected or noninfected treatments were analyzed using Proc Means program (SPSS 19.0, IBM, Armonk, NY, USA). Differences were compared using a *t*-test (PRT program) at significance levels of *p* < 0.05 or *p* < 0.01.

## 3. Results

### 3.1. Endosymbiont Disruption in Pea Aphids

**Figure 1:**
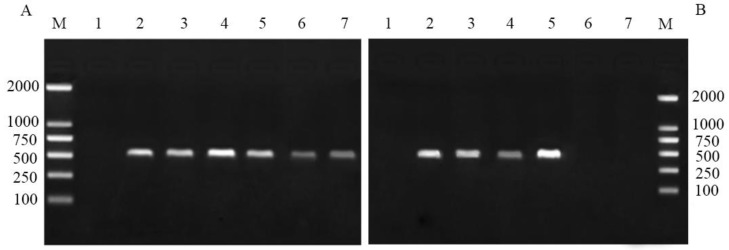
(**A**) PCR detection of *Buchnera aphidicola* in pea aphids on day 6 after antibiotic treatment. (**B**) PCR detection of *Buchnera* in pea aphids on day 11 after antibiotic treatment. M: molecular weight marker, line 1: negative control of ddH_2_O, line 2: antibiotics of chloraomycetin, 3: penicillin-GK salt, 4: streptomycin sulfate, 5: chlortetracycline HCl, 6: rifampicin, 7: oxytetracycline HCl.

The disruption of primary endosymbiont *Buchnera* in pea aphids was detected by PCR with specific primers. The results showed that *Buchnera* in the pea aphid nymphs treated with the six antibiotics were all positive during initial 6 days (including the 2-day feeding on antibiotic solution cultured plants) (Figure 1A). This means that all of the six antibiotics failed to remove *Buchnera* during this period. However, the PCR detection on day 11 showed that *Buchnera* was negative in the rifampicin and terramycin treatments, but the mitochondrial COI reference gene (555 bp fragment) in these two treatments was positive (figure not shown), indicating that the extracted DNA in all the treatments were of high quality. Moreover, in the other four antibiotic treatments, *Buchnera* was still positive (Figure 1B), which indicated that rifampicin and terramycin can successfully disrupt the *Buchnera* endosymbiont of pea aphid nymphs after 11 days. 

### 3.2. Effects of Buchnera Disruption on the Growth of Pea Aphid

The body size and weight of pea aphids on day 11 were compared between the *Buchnera*-infected and *Buchnera*-disrupted individuals. Body size and weight were significantly affected by the disruption of *Buchnera*. A healthy pea aphid of 11-days age was on average 3333.26 µm in length and 1411.80 µm in width, while a *Buchnera*-disrupted aphid was 1788.74 µm in length and 705.23 µm in width, almost half the size of the healthy aphid. The average weight of a healthy aphid was 3.53 mg, while a *Buchnera*-disrupted aphid was 1.24 mg, about a 65% weight reduction compared to the healthy aphid. Furthermore, when pea aphids were treated with antibiotics leading to *Buchnera* disruption, pea aphids lost their ability to reproduce (Table 1).

### 3.3. Quantification of the Dynamic Changes of Proteins

**Figure 2:**
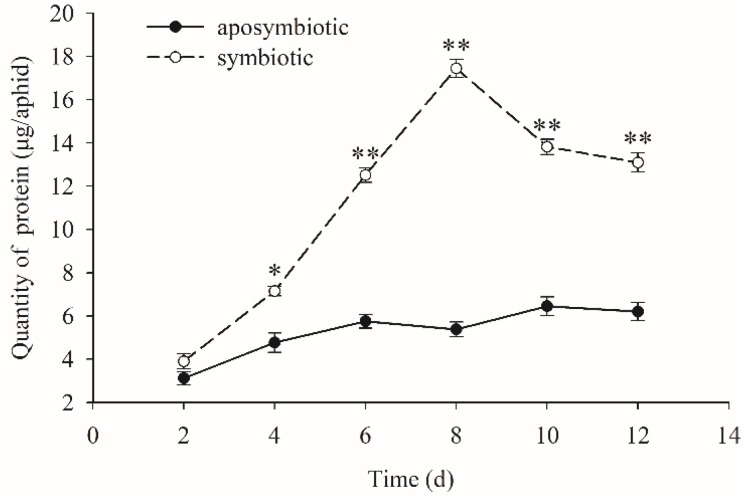
Dynamic changes of protein content in pea aphids that were *Buchnera* absent or infected. Black dot: rifampicin-treated aphid; Clear circle: healthy control aphid. The black dot or clear circle and error bars were the mean ± standard error. The star “*” means significant differences between the antibiotic-treated and control aphids at 0.05, the double star “**” means significant differences between the antibiotic-treated and control aphids at 0.01. (*t* = 2.344, *df* = 16, *p* = 0.041 on day 4; *t* = 5.991, *df* = 16, *p* < 0.01 on day 6; *t* = 7.155, *df* = 16, *p* < 0.01 on day 8; *t* = 5.321, *df* = 16, *p* < 0.01 on day 10; *t* = 3.765, *df* = 16, *p* ≤ 0.01 on day 12).

When the *Buchnera* endosymbiont was disrupted, the quantity of protein content increased slowly with the growth of the pea aphid host, while that of a healthy aphid in control experiments obviously increased with the growth of the host and reached the maximum quantity on 8-days age, approximately 18 µg/aphid (Figure 2). This was three times higher than that of an antibiotic-treated aphid. There were also significant differences between the protein contents of *Buchnera*-absent and *Buchnera*-infected aphids during days 4–12 (*t* = 2.344, *df* = 16, *p* = 0.041 on day 4; *t* = 5.991, *df* = 16, *p* < 0.01 on day 6; *t* = 7.155, *df* = 16, *p* < 0.01 on day 8; *t* = 5.321, *df* = 16, *p* < 0.01 on day 10; *t* = 3.765, *df* = 16, *p* ≤ 0.01 on day 12).

### 3.4. Quantification for the Dynamic Changes of Carbohydrates

**Figure 3:**
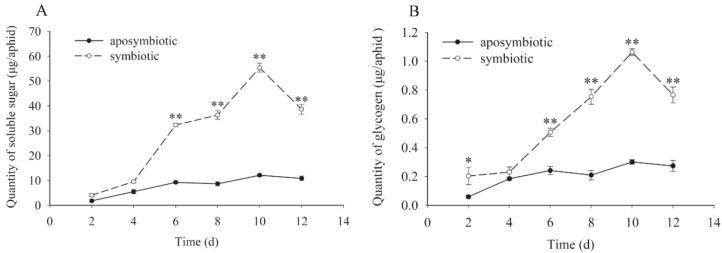
Dynamic changes of soluble sugars and glycogen in pea aphids that were *Buchnera* absent or infected. Black dot: rifampicin-treated aphid; Clear circle: healthy control aphid. The black dot or clear circle with error bars were mean ± standard error. The star “*” means significant differences between the antibiotic-treated and control aphids at 0.05, the double star “**” means significant differences between the antibiotic-treated and control aphids at 0.01. (**A**) Dynamic changes of soluble sugars (*t* = 4.508, *df* = 16, *p* < 0.01 on day 6; t = 14.486, *df* = 16, *p* < 0.01 on day 8; *t* = 13.740, *df* = 16, *p* < 0.01 on day 10; *t* = 14.923, *df* = 16, *p* < 0.01 on day 12). (**B**) Dynamic changes of glycogen (*t* = 2.346, *df* = 16, *p* = 0.041 on day 2; *t* = 4.083, *df* = 16, *p* < 0.01 on day 6; *t* = 8.673, *df* = 16, *p* < 0.01 on day 8; *t* = 26.878, *df* = 16, *p* < 0.01 on day 10; *t* = 7.381, *df* = 16, *p* < 0.01 on day 12).

Similar to the trend for protein, both the quantity of soluble sugar and glycogen (Figure 3) in *Buchnera*-disrupted aphids increased slowly with the aphid host growth. In contrast, that of healthy aphids in control experiments increased greatly over the experimental time period. The maximum quantity for sugar and glycogen in control aphids and treatment were observed at 10 days of age, with approximately 58 µg/aphid and 1.2 µg/aphid, respectively. Significant differences were recorded between the antibiotic-treated and control aphids in days 6–12 in both soluble sugar (t = 4.508, *df* = 16, *p* < 0.01 on day 6; t = 14.486, *df* = 16, *p* < 0.01 on day 8; t = 13.740, *df* = 16, *p* < 0.01 on day 10; t = 14.923, *df* = 16, *p* < 0.01 on day 12) and glycogen (t = 2.346, *df* = 16, *p* = 0.041 on day 2; t = 4.083, *df* = 16, *p* < 0.01 on day 6; t = 8.673, *df* = 16, *p* < 0.01 on day 8; t = 26.878, *df* = 16, *p* < 0.01 on day 10; t = 7.381, *df* = 16, *p* < 0.01 on day 12) detection.

### 3.5. Quantification of the Dynamic Changes to Lipids

**Figure 4:**
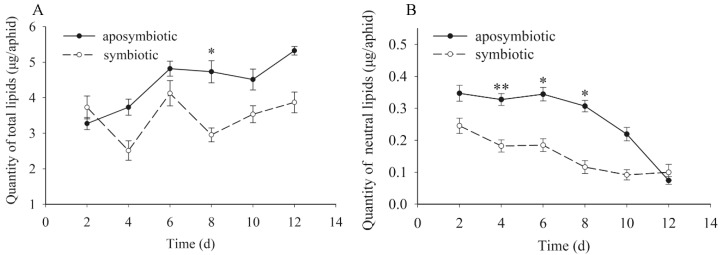
Dynamic changes of total lipids and neutral fats in pea aphids that were *Buchnera* absent or infected. Black dot: rifampicin-treated aphid; Clear circle: healthy control aphid. The star “*” means significant differences between the antibiotic-treated and control aphids, the double star “**” means significant differences between the antibiotic-treated and control aphids at 0.01. The black dot or clear circle with error bars were the mean ± standard error. (**A**) Dynamic changes of total lipids (*t* = 3.113, *df* = 16, *p* = 0.011 on day 8). (**B**) Dynamic changes of neutral fats (*t* = 3.373, *df* = 16, *p* < 0.01 on day 4; *t* = 2.539, *df* = 16, *p* = 0.029 on day 6; *t* = 2.278, *df* = 16, *p* = 0.046 on day 8).

The trends of total lipids and neutral fat showed that dynamic changes were also similar to the protein and carbohydrates (Figure 4). However, contrary to both of them, the qualities of total lipid and neutral fat in *Buchnera*-disrupted pea aphids were higher than that of healthy control aphids. During the experimental period, the quantity of total lipid waved gently and showed an increasing trend in both the antibiotic-treated and control aphids in general. Similarly, the quantity of neutral fats in *Buchnera*-disrupted aphids was distinctly higher than that of healthy aphids (t = 3.373, *df* = 16, *p* < 0.01 on day 4; t = 2.539, *df* = 16, *p* = 0.029 on day 6; t = 2.278, *df* = 16, *p* = 0.046 on day 8) but different than the trend of total lipids. The quantity of neutral fat in both types of aphid showed an obvious decrease during the experimental period. 

## 4. Discussion

The important roles of primary endosymbionts to their insect hosts are well studied. In previous studies, aphid interactions with *Buchnera* have been investigated from many different perspectives, including the nutritional and metabolic complementation within the aphid body [10,29,35,36,37,38]. However, relatively little is known about the nutritional dynamic change in aphids when *Buchnera* is absent. In the current study, the efficiencies of six antibiotics in disrupting *Buchnera* within pea aphids were compared. By using PCR specific amplification, rifampicin was revealed to be one of the most efficient antibiotics that can disrupt *Buchnera* in the pea aphid. Our screening results were consistent with other researchers, such as Machado-Assefh et al. [29] and Zhang et al. [38]. Adams et al. [39] found that the fresh weight of a rifampicin-treated pea aphid was 0.82 mg/aphid, which was only about 28% of the weight of control pea aphids. Zhang et al. [38] also revealed that elimination of *Buchnera* using the antibiotic rifampicin significantly reduced the body mass of the English grain aphid, *Sitobion avenae*. In our current study, the disruption of *Buchnera* in a pea aphid significantly reduced both body size and body mass, thus supporting the findings of Adams et al. [39] and Zhang et al. [38].

Previous studies have revealed that *Buchnera* is involved in biosynthesis of more than 10 essential amino acids required by an aphid host [40,41]. Thus, the disruption of *Buchnera* can explain why the protein contents of the pea aphid were significantly lower than that of the healthy control aphids in the current study. In addition, a previous study has shown that the reproduction ability of pea aphids is correlated with protein content: the greater protein content, the higher the reproduction ability [42]. Ahsaei et al. [43] also reported that ecological adaptation, especially the fertility of a pea aphid, was closely related to the protein contents in its body. Moreover, our research showed that disruption of *Buchnera* infection in aphids led to a failure in offspring production. Therefore, we deduced that the disruption of *Buchnera* directly led to the reduction of protein contents, which further caused the reproductive declension of the pea aphid host. However, this needs further clarification.

Our study results also showed that both soluble sugar and glycogen contents decreased with the disruption of *Buchnera* in the pea aphid. It has been reported that the function of soluble sugar is to offer energy for the host’s muscles when an insect is walking or escaping [44]. Thus, the disruption of *Buchnera* first leads to a soluble sugar decrease and so weakens the behaviour of the pea aphid host. Although we did not investigate the behaviour of the pea aphid in the current study, a previous study has reported that the disruption of *Buchnera* in the aphid *Myzus persicae* negatively affected the feeding behaviour of the aphid and further delayed its host plant acceptance [29]. 

An interesting finding from the current study was that, as *Buchnera* was disrupted, the quantity of total lipids and neutral fats was significantly higher than that of control aphids. This may be due to the fact that the final transformation of extra soluble sugar is for the synthesis of lipid [45]. The toxicity of antibiotics may directly influence aphids’ weight and body size as well as the intestinal microorganism composition and their function, and when *Buchnera* is disrupted by rifampicin, this may result in metabolic problems imposed by the loss of the endogenous supply of essential amino acids in the pea aphid. The loss of an endogenous supply of essential amino acids has multiple secondary effects on aphid metabolism. So, the effect of metabolism was the result of the combined action of the loss of *Buchnera* and antibiotic toxicity, but the loss of *Buchnera* has a great influence on the metabolism of aphids.

In summary, the effects of *Buchnera* disruption may lead to distinctly dynamic changes of nutrition in its pea aphid host, including protein, soluble sugar, glycogen, total lipid, and neutral fat. All the changes may cause the energy levels of the aphid host to vary. An energy analysis with regards to the disruption of *Buchnera* in the pea aphid was performed in a previous study [46]. One 6-day-old pea aphid was used for analysis between rifampicin-treated and healthy control aphids. Here, the energy stored in protein and carbohydrates declined 68.64% and 21.94%, respectively, although energy stored in total lipids increased 60.08%. However, the total energy stored in one aphid after 6 days declined 58.12% in total. Since the energy decrease weakens the aphid’s behaviour, especially in regards to foraging and crop damage, endosymbiont disruption could form a novel strategy for hemipteran pest control. 

## 5. Conclusions

In conclusion, this study confirmed that the function of *Buchnera* is irreplaceable in the pea aphid. Compared to healthy control aphids, the body masses of pea aphids were reduced, the reproduction ability was lost, and the body length and width were also significantly decreased after function of *Buchnera* was disrupted by using the antibiotic rifampicin. The quantity of proteins, soluble sugars, and glycogen significantly decreased, but the quantity of total lipid and neutral fat increased in aposymbiotic pea aphids.

## Figures and Tables

**Table 1:** The effects of *Buchnera* disruption on the body mass and fecundity of the pea aphid.

	Symbiotic Pea Aphids	Aposymbiotic Pea Aphids	
Length (μm)	3333.26 ± 70.19	1788.64 ± 44.07 *	t = 28.314, *df* = 58, *p* < 0.05
Width (μm)	1411.80 ± 37.30	705.23 ± 32.59 *	t = 16.447, *df* = 58, *p* < 0.05
Weight (mg)	3.525 ± 0.125	1.238 ± 0.132 *	t = 27.691, *df* = 58, *p* < 0.05
Number of offspring	60.57 ± 1.98	0 *	t = 121.911, *df* = 58, *p* < 0.01

The columns were mean ± standard error. The star “*” means significant differences between the antibiotic treated and control aphid at 0.05.
